# Contributions of the N-terminal flanking residues of an antigenic peptide from the Japanese cedar pollen allergen Cry j 1 to the T-cell activation by HLA-DP5

**DOI:** 10.1093/intimm/dxad024

**Published:** 2023-09-05

**Authors:** Seisuke Kusano, Sho Ueda, Daisuke Oryoji, Aya Toyoumi, Akiko Hashimoto-Tane, Hiroyuki Kishi, Hiroshi Hamana, Atsushi Muraguchi, Hui Jin, Hisashi Arase, Hiroko Miyadera, Reiko Kishikawa, Yasunobu Yoshikai, Hisakata Yamada, Ken Yamamoto, Yasuharu Nishimura, Takashi Saito, Takehiko Sasazuki, Shigeyuki Yokoyama

**Affiliations:** RIKEN Structural Biology Laboratory, Yokohama 230-0045, Japan; RIKEN Cluster for Science, Technology and Innovation Hub, Yokohama 230-0045, Japan; Institute for Advanced Study, Kyushu University, Fukuoka 812-8582, Japan; Institute for Advanced Study, Kyushu University, Fukuoka 812-8582, Japan; Institute for Advanced Study, Kyushu University, Fukuoka 812-8582, Japan; RIKEN Center for Integrative Medical Sciences, Yokohama 230-0045, Japan; Department of Immunology, Faculty of Medicine, Academic Assembly, University of Toyama, Toyama 930-0194, Japan; Department of Immunology, Faculty of Medicine, Academic Assembly, University of Toyama, Toyama 930-0194, Japan; Department of Immunology, Faculty of Medicine, Academic Assembly, University of Toyama, Toyama 930-0194, Japan; Department of Immunochemistry, Research Institute for Microbial Diseases, Osaka University, Osaka 565-0871, Japan; Department of Immunochemistry, Research Institute for Microbial Diseases, Osaka University, Osaka 565-0871, Japan; Laboratory of Immunochemistry, WPI Immunology Frontier Research Center, Osaka University, Osaka 565-0871, Japan; Department of Human Genetics, Graduate School of Medicine, The University of Tokyo, Tokyo 113-0033, Japan; Research Institute, National Center for Global Health and Medicine, Chiba 272-8516, Japan; Department of Medical Genetics, Institute of Medicine, University of Tsukuba, Tsukuba 305-8575, Japan; Department of Allergology, The National Hospital Organization Fukuoka National Hospital, Fukuoka 811-1394, Japan; Medical Institute of Bioregulation, Kyushu University, Fukuoka 812-8582, Japan; Medical Institute of Bioregulation, Kyushu University, Fukuoka 812-8582, Japan; Department of Medical Biochemistry, Kurume University School of Medicine, Kurume 830-0011, Japan; Department of Immunogenetics, Graduate School of Medical Sciences, Kumamoto University, Kumamoto 860-8556, Japan; RIKEN Center for Integrative Medical Sciences, Yokohama 230-0045, Japan; Institute for Advanced Study, Kyushu University, Fukuoka 812-8582, Japan; RIKEN Structural Biology Laboratory, Yokohama 230-0045, Japan; RIKEN Cluster for Science, Technology and Innovation Hub, Yokohama 230-0045, Japan

**Keywords:** immunogenicity, Japanese cedar pollinosis, peptide flanking region

## Abstract

Cry j 1 is a major allergen present in Japanese cedar (*Cryptomeria japonica*) pollens. Peptides with the core sequence of KVTVAFNQF from Cry j 1 (‘pCj1’) bind to HLA-DP5 and activate Th2 cells. In this study, we noticed that Ser and Lys at positions −2 and −3, respectively, in the N-terminal flanking (NF) region to pCj1 are conserved well in HLA-DP5-binding allergen peptides. A competitive binding assay showed that the double mutation of Ser(–2) and Lys(–3) to Glu [S(P–2)E/K(P–3)E] in a 13-residue Cry j 1 peptide (NF-pCj1) decreased its affinity for HLA-DP5 by about 2-fold. Similarly, this double mutation reduced, by about 2-fold, the amount of NF-pCj1 presented on the surface of mouse antigen-presenting dendritic cell line 1 (mDC1) cells stably expressing HLA-DP5. We established NF-pCj1-specific and HLA-DP5-restricted CD4^+^ T-cell clones from HLA-DP5 positive cedar pollinosis (CP) patients, and analyzed their IL-2 production due to the activation of mouse TG40 cells expressing the cloned T-cell receptor by the NF-pCj1-presenting mDC1 cells. The T-cell activation was actually decreased by the S(P–2)E/K(P–3)E mutation, corresponding to the reduction in the peptide presentation by this mutation. In contrast, the affinity of NF-pCj1·HLA-DP5 for the T-cell receptor was not affected by the S(P–2)E/K(P–3)E mutation, as analyzed by surface plasmon resonance. Considering the positional and side-chain differences of these NF residues from previously reported T-cell activating sequences, the mechanisms of enhanced T-cell activation by Ser(–2) and Lys(–3) of NF-pCj1 may be novel.

## Teaser

Flanking residues enhance the presentation of allergenic peptides

## Introduction

In Japan, the most prevalent allergen is Japanese cedar (*Cryptomeria japonica*) pollen (1). The number of Japanese cedar pollinosis (CP) patients in Japan has rapidly increased since the 1960s (2), and is now more than 25 million (3). The two major allergens, Cry j 1 (4) and Cry j 2 (5), and other allergens, such as Cry j 3 (6) and Cry j 4 (6), from Japanese cedar pollen react with immunoglobulin E (IgE) antibodies from the pollinosis patients (6, 7). Cry j 1 and Cry j 2 are 41–46 kDa and 45 kDa basic glycoproteins, respectively, and share 70–80% amino acid sequence identity (8). They were identified as a pectate lyase (isozyme) (9) and a polymethylgalacturonase (enzyme) (10), respectively. Cry j 1 and Cry j 2 are similarly abundant in the pollen. The former exists in Ubisch bodies on the surface of the pollen, while the latter is on the inside, within the cytoplasm and starch granules (11). Therefore, Cry j 1 is much more abundant than Cry j 2 in the patients’ nasal discharge.

By analyzing the statistical association between Japanese CP and class II human major histocompatibility complex (MHC-II) alleles, we found that about 80% of the patients possess the human MHC-II protein HLA-DP5 (DPA1*02:02 and DPB1*05:01) (12). We identified an antigenic peptide that induces HLA-DP5-restricted Th2 cells, by testing thirty-eight 13-residue peptides spanning the entire length of Cry j 1. The elucidated nine-residue ‘core’ sequence for the HLA-DP5-restricted Th cells is 214-KVTVAFNQF-222 of Cry j 1 (12), designated hereafter as ‘pCj1’. We determined the crystal structure of the extracellular region of the HLA-DP5 (DPA1*02:02 and DPB1*05:01) heterodimer in complex with pCj1, at 2.4 Å resolution (13). The pCj1 peptide is accommodated in the peptide-binding groove (PBG) of the HLA-DP5 extracellular region, and is sequence-specifically recognized. In particular, the negatively charged P1 pocket characteristically recognizes the Lys side-chain at the first anchoring position in the peptide sequence (13).

Like other class-II molecules, HLA-DP5 can bind longer peptides than the nine-residue core sequence pCj1, such as the 13-residue peptides described above (12), and its PBG is ‘open’ at both ends (13), allowing the protrusion of the peptide flanking residues (PFRs) on both sides of the core sequence. The PFRs reportedly contribute to the high efficiencies of T-cell activation by the peptide-bound MHC-II molecules (pMHCs) (14). Structural studies have indicated the mechanisms by which the PFRs contribute to the T-cell activation. In some cases, the affinity of the peptide for the MHC-II molecule is increased by the interaction of the N-terminal flanking (NF) residue at position −1 (P–1), next to the P1 position of the core sequence, with the MHC-II molecule (15, 16). In contrast, in several other cases, an Arg residue in the C-terminal flanking (CF) region and a Gly/Lys residue in the NF region are considered to increase the affinity of the pMHC-II molecule for the T-cell receptor (TCR), through a direct interaction between the specific PFR residue and the TCR molecule (17–19).

In the present study, we established HLA-DP5-restricted T-cell clones that are specific to the 13-residue peptide consisting of the four-residue NF region and the nine-residue core pCj1 [DKSMKVTVAFNQF (‘NF-pCj1’), where the four NF residues are underlined]. Characteristically, Ser and Lys at positions −2 and −3 in the NF sequence of NF-pCj1 are well conserved in other HLA-DP5-binding epitope peptides of pollen allergens. A comparison of the S(P–2)E/K(P–3)E mutant with the wild-type (WT) peptide revealed that the Ser and Lys residues increase the binding affinity of the peptide for HLA-DP5, the amount of the peptide displayed on the surface of antigen-presenting dendritic cells, and the T-cell activation by HLA-DP5. In contrast, the affinity of NF-pCj1•HLA-DP5 for the TCRs was not affected by the S(P–2)E/K(P–3)E mutation. Therefore, the mechanisms by which the NF residues contribute to the T-cell activation are different from those reported previously for other antigenic peptides.

## Methods

### Peptide competition assay

A biotin-conjugated HA1 peptide, biotin-ENERTLDFHDSNVKN, was chemically synthesized, with the biotin label at the N-terminus, for peptide competition assay. Expression plasmids containing the full-length cDNAs encoding DPA1*02:02, DPB1*05:01, and GFP were transiently transfected into HEK293 cells. After 24 h, the HLA-DP5-transfected cells were cultured for an additional 24 h in 96-well flat-bottom plates (5.0 × 10^4^ cells per well) in the presence of the biotin-conjugated HA1 peptide (10 μg ml^−1^) and various concentrations of the chemically synthesized NF[WT]-pCj1 or NF[S(P–2)E/K(P–3)E]-pCj1 peptides (Toray) (1–300 μg ml^−1^) in culture medium (DMEM containing 10% Fetal Bovine Serum). These transfectants were then stained with allophycocyanin-conjugated streptavidin (Jackson ImmunoResearch) and analyzed by BD FACSCalibur cell cytometry (BD Biosciences). Specific binding of the biotin-conjugated HA1 peptide to HLA-DP5 was calculated by subtracting the mean fluorescence intensities (MFIs) of the GFP-negative HLA-DP non-expressing population from those of the GFP-positive HLA-DP-expressing population. The half maximal inhibitory concentration (IC_50_) was calculated from the specific binding. Similarly, peptide competition assays were performed with not only NF[WT]-pCj1 and NF[S(P–2)E/K(P–3)E]-pCj1 peptides but also NF[S(P–2)E]-pCj1 and NF[K(P–3)E]-pCj1 peptides (Toray). It may be possible that the N-terminal extension and/or amino acid substitution in the NF affected not only HLA-DP5-binding but also local concentration of intact peptides. We think that the local concentration of the peptide is not much affected by the mutations because there coexist large amounts of other peptides and proteins.

### Stable cell lines expressing HLA-DP5 for peptide-display and IL-2 production assays

The full-length cDNAs encoding the α and β chains of HLA-DP5 (DPA1*02:02 and DPB1*05:01, respectively) were inserted into the retroviral vectors pMXs-Puro and pMXs-IB (20), respectively, with an *Eco*RI site at the 5ʹ terminus and a *Not*I site at the 3ʹ terminus. To generate the retroviruses containing pMXs-Puro/DPA1 and pMXs-IB/DPB1, approximately 0.5–1.0 × 10^6^ PLAT-A (21) or PLAT-E (22) cells were transfected with 1.5 μg plasmid and 4.5 μl Fu-GENE reagent (Roche Diagnostics), according to the manufacturer’s instructions. The DPA1-stable cells were established by the transduction of mouse antigen-presenting dendritic cell line 1 (mDC1) cells with a retrovirus containing pMXs-Puro/DPA1 and selection with puromycin (5 μg ml^−1^). The DPA1-stable cells were subsequently transduced with pMXs-IB/DPB1 and selected with blasticidin (5 μg ml^−1^), to obtain DPA1- and DPB1-stable cells. Site-directed mutagenesis was performed with a QuikChange II kit (Agilent Technologies), according to the manufacturer’s instructions. The cell-surface expression levels of HLA-DP were analyzed with an EPICS-XL (Beckman Coulter) or BD FACSCalibur (BD Biosciences) flow cytometer. HLA-DP was stained with anti-HLA-DP (B7/21) (Leinco Technologies), and mouse IgG3 (6A3) (MBL), as the isotype control, was stained with phycoerythrin-conjugated anti-mouse IgG3 (Santa Cruz Biotechnology). mDC1 cells with certain uniform levels of HLA-DP5 expression were collected and used for the quantitative assays of cell-surface peptide-display and IL-2 production.

### Fluorescent peptides

The fluorescent versions of NF-pCj1 with the WT or S(P–2)E/K(P–3)E mutant NF were chemically synthesized (Cosmo Bio) to consist of NF-pCj1, a three-residue peptide spacer (GPN), a two-ethylene glycol unit spacer (EG_2_), and a TAMRA dye molecule in this order, as NF[WT]-pCj1–TAMRA (DKSMKVTVAFNQF-GPN-EG_2_-TAMRA) and NF[S(P–2)E/K(P–3)E]-pCj1TAMRA (DEEMKVTVAFNQF-GPN-EG_2_-TAMRA), respectively. These fluorescent peptides were dissolved in DMSO, and stored as 5 mM (~12 mg ml^−1^) stock solutions at − 20°C.

### Peptide-display assay

The parent mDC1 cells without HLA-DP5 expression (HLA-DP5[−]), and the HLA-DP5-expressing mDC1 cells, HLA-DP5[WT], were diluted with DMEM to 1.0 × 10^6^ cells ml^−1^. The cells were cultured in 96-well round-bottom plates (0.5 × 10^5^ cells per well), and the fluorescent peptides were added at various concentrations (0.25–4 μM) by dilution with the medium. After an incubation for 3.5 h at 37°C, the cells were washed with cold PBS. The fluorescent peptides assimilated by the cells were recognized with an anti-TAMRA mAb (5G5, Abcam) labeled with Alexa Fluor 647 (Zeon Alexa Fluor 647 Mouse IgG1 Labeling Kit, Thermo Fisher Scientific) by an incubation for 25 min at 4°C, according to the manufacturer’s instructions. The cells were then washed with cold DMEM. Thereafter, the cells were stained with Hoechst 33342 (diluted 1: 1000, at 4°C) (Thermo Fisher Scientific), and analyzed by a flow cytometer (BD LSRFortessa X-20, BD Biosciences) for the fluorescent intensity in the whole cell (TAMRA, excited at 561 nm) and that on the cell surface (Alexa647, excited at 640 nm). The mean fluorescent intensity (MFI) of either TAMRA or Alexa647 for each sample was corrected by calculating the difference between the MFI value in the presence of the TAMRA-conjugated NF-pCj1 and that in its absence. The total amount of the TAMRA-conjugated peptide displayed on the cell surface for the HLA-DP5-expressing mDC1s was estimated by subtracting the values for the parent mDC1s from the corrected MFI values. All data were analyzed with the FlowJo software (BD Biosciences).

### Preparation of peripheral blood mononuclear cells

Peripheral blood was obtained from HLA-DP5^+^ CP patients. Peripheral blood mononuclear cells (PBMCs) were isolated by a density gradient technique, using Ficoll-Paque PLUS. The research protocol for collecting and using PBMCs from allergic patients was approved by the Institutional Review Board of Kyushu University.

### Cloning of antigen-specific CD4^+^ T cells

Cloning of CD4^+^ T cells was performed as described previously (23). A single, viable (7AAD^–^), CD4^+^, CFSE^dim^ cell was sorted into each well of a 96-well plate, which contained 100 μl of culture medium with feeder cells and cytokines, as described below. Irradiated (30 Gy) peptide-pulsed PBMCs (1.0 × 10^5^) isolated from unrelated DPB1*05:01^+^ donors were used as feeder cells in the cloning plates. Pooled human serum (5%) and IL-2 (100 U ml^−1^) (Peprotech) were added at the indicated final concentrations to AIM-V medium (Life Technologies). Cells were fed every 7 days with fresh cytokine and peptide-pulsed PBMCs. Growing clones were identified by visual inspection, expanded into ~24 six-well plates, and then tested for antigen specificity by ^3^H-thymidine incorporation and cytokine production assays, using HLA-DP5 transfected K562 cells as antigen-presenting cells (APCs). HLA-DP5-K562 cells were kindly provided by Dr H. Sugiyama, Osaka University. CD4^+^ T-cell clones were stained with a PE-conjugated anti-human CD4 monoclonal antibody (mAb), and single cells were sorted into MicroAmp reaction tubes. The cDNAs encoding TCR α and β were amplified from single cells, inserted into the pMX vector (generously provided by Dr T. Kitamura, University of Tokyo), and then transfected into the packaging cell line PLAT-E (kindly supplied by Dr T. Kitamura, University of Tokyo), as described previously (22). The culture supernatant containing the recombinant retroviruses was added to TG40 cells transfected with the human CD4 gene (CD4^+^ TG40), while the original TG40 cells do not express CD4. TCR expression was examined by monitoring the expression of mouse CD3ε by flow cytometry.

### NF-pCj1-specific and HLA-DP5-restricted T-cell clones

We first obtained NF-pCj1-specific and HLA-DP5-restricted T-cell clones, as follows. Analyses of PBMCs from patients with CP demonstrated the presence of CD4^+^ T cells that proliferated upon the recognition of NF-pCj1 presented by HLA-DP5, and a representative analysis is shown in Fig. 4A. Using the proliferated CD4^+^ T cells from a CP patient (SU), we established NF-pCj1-specific and HLA-DP5-restricted T-cell clones (SU3–2 and SU3–3) in the presence of HLA-DP5^+^ PBMCs. These responses were almost completely blocked by a mAb against HLA-DP, but not by mAbs against either DR or DQ, confirming that these T-cell clones are DP5-restricted. We also produced T-cell clones (NM-4 and NM-7) from another CP patient (NM), which specifically proliferated in response to NF-pCj1 and are restricted by HLA-DP5. Upon stimulation with NF-pCj1 in the presence of HLA-DP5-transfected K562 cells, SU3–2, SU3–3, NM-4, and NM-7 produced IL-5, IL-10, and IL-13, indicating that these clones have the characteristics of Th2 cells. For further analyses, we cloned the TCRα and TCRβ genes from SU3–2, SU3–3, NM-4, and NM-7, and established TCRαβ-expressing CD4^+^ TG40 cells [TCR(SU3–2)-TG40, TCR(SU3–3)-TG40, TCR(NM-4)-TG40, and TCR(NM-7)-TG40]. These TCR-transfected TG40 cells responded to NF-pCj1, but not to pCj1, as measured by the CD137^+^ cell number in the presence of the HLA-DP5^+^ PBMCs.

### TCR-transfected cell line

T-cell lines were single-cell sorted into 96-well PCR plates by using a cell sorter (BD FACSAria II, BD Biosciences), and were stored at −80°C until the RT-PCR reaction. The TCRα and β cDNAs were amplified by single-cell RT-PCR, and the PCR products were examined by direct sequencing. The TCR repertoire was analyzed with the IMGT/V-Quest tool (https://www.imgt.org/). The TCRβ and α cDNAs were linked with a self-cleaving P2A fragment, and inserted into the pMXs − IRES − GFP retroviral vector (Cell Biolabs) by using Gibson Assembly Master Mix (New England Biolabs), according to the manufacturer’s instructions. The nucleotide sequence of the plasmid DNA was confirmed by DNA sequencing. The constructed plasmid vector, pMXs/TCRβ−P2A − TCRα/−IRES − GFP, was then transfected into Phoenix A cells (kindly provided by Dr Garry Nolan, Stanford University) with FuGENE 6 (Roche). The culture supernatant was collected 72 h after the transfection. Human CD4^+^ TG40 cells were infected with the recombinant retroviruses by using retronectin (Takara Bio), according to the manufacturer’s instructions.

### CFSE assay to detect T-cell proliferation

The CFSE assay was performed as described previously (24). Briefly, PBMCs (1.0 × 10^7^ cells ml^−1^ in PBS) were incubated for 10 min with 5 μM CFSE (Molecular Probes). Staining was terminated by adding PBS containing 5% pooled human serum, and after the cells were washed three times in PBS containing 1% pooled human serum, they were resuspended in culture medium (RPMI-1640 containing 5% pooled human serum) at 2.0 × 10^6^ cells ml^−1^. Stained cells (2.0 × 10^6^ cells well^−1^, 1 ml) were cultured in 24-well plates with medium alone, pCj1 (final concentration, 10 μg ml^−1^), or NF-pCj1 (10 μg ml^−1^). After 7 days, the cells treated with each antigen were pooled, washed in PBS, and stained with anti-human CD4-allophycocyanin (IgG1, clone RPA-T4, BioLegend) and 7-amino-actinomycin D (7-AAD) (BD Pharmingen). Antigen-specific, viable CD4^+^ T cells were identified as 7AAD^−^, CD4^+^, CFSE^dim^ cells, and the proliferation levels of these antigen-specific CD4^+^ T cells were examined in each experiment.

### ^3^H-thymidine incorporation assay to detect T-cell proliferation

T-cell clones were tested for antigen specificity, using the ^3^H-thymidine incorporation assay. The APCs were HLA-DP5-positive allogenic PBMCs, which had been thawed and irradiated (30 Gy). Each clone (3.0 × 10^4^ cells per well) was stimulated in duplicate with APCs (1.0 × 10^5^ cells per well) that had been pulsed with each of the peptides. Cultures were set up in 96-well round-bottom plates with 200 μl RPMI-1640 containing 5% human serum. After two days, ^3^H-thymidine (1 μCi per well) was added for 18 h. The cells were then harvested and the incorporated radioactivity was measured by β-scintillation counting. Clones that showed a stimulation index (SI, cpm with antigen/cpm without antigen) of >3 were considered to be antigen-specific. To determine the HLA molecules involved in antigen presentation, the proliferation of the CD4^+^ T-cell clones was blocked by adding an anti-HLA-DR mAb (L243, BioLegend), an anti-HLA-DP mAb (B7/21, Abcam), or an anti-human HLA-DQ mAb (SPV-L3, Abcam). All mAbs were used at a final concentration of 10 μg ml^−1^.

### IL-2 production assay

HLA-DP5-expressing mDC1 cells (5.0 × 10^3^ cells per well) were cultured with NF-pCj1 for 3 h in 96-well flat-bottom plates. TCR-TG40 cells (3.0 × 10^4^ cells per well) were added to the culture medium, and after 18 h the IL-2 production in the culture supernatant was measured using an ELISA kit (Thermo Fisher Scientific), according to the manufacturer’s instructions.

### Preparation of the NF-pCj1–HLA-DP5^ec^ molecule

The NF-pCj1–HLA-DP5^ec^ molecule was prepared in the same manner as the pCj1–HLA-DP5^ec^ molecule (13), except that the NF-pCj1 peptide consists of thirteen residues, the NF residues [DKSM] and the core residues of pCj1 [KVTVAFNQF]. Briefly, the NF-pCj1–HLA-DP5^ec^ and pCj1–HLA-DP5^ec^ samples for the surface plasmon resonance (SPR) analysis, consisting of the HLA-DP5α protein and the NF-pCj1–HLA-DP5β fusion protein, were synthesized by the cell-free protein synthesis method with an *Escherichia coli* cell extract (25, 26), and purified by affinity and size exclusion chromatography (SEC).

### Preparation of the TCR^ec^ molecule for SPR

DNA fragments encoding the extracellular regions of the TCR(SU3–3) α chain (residues 1–209) and the TCR(SU3–3) β chain (residues 1–245) with the mutations T163C and S172C/C190A, respectively, to form an artificial interchain disulfide bond, were chemically synthesized according to the *E. coli* codon usage. These DNA fragments were subcloned together with those encoding the N11-SUMO tag at the N-terminus and the TEV protease cleavage site followed by hexahistidine and FLAG tags, respectively, at the C-terminus, into the TA vector pCR2.1TOPO (Invitrogen). The tagged α and β chains of TCR(SU3–3)^ec^ were co-expressed in the presence of the disulfide isomerase DsbC and GSH/GSSG, by the *E. coli* cell-free protein synthesis method operated in the large-scale dialysis mode (25, 26). The prepared complex of the TCR(SU3–3)^ec^ with tagged α and β chains was purified by anti-FLAG M2 affinity agarose gel chromatography (Sigma). The tags were cleaved by digestions with SUMO and TEV proteases overnight at room temperature, and the N11-SUMO and hexahistidine tags were removed with Ni-NTA agarose (Qiagen). Finally, the fraction containing the TCR(SU3–3)^ec^ αβ molecule was subjected to SEC on a Superdex 200 10/300 GL (GE Healthcare) column, and the fractions exhibiting the bands of the α and β chains of TCR(SU3–3)^ec^ αβ in the SDS-PAGE analysis were collected, concentrated to 10 μM, and utilized in the SPR analysis.

### SPR analysis

The ligands, NF-pCj1–HLA-DP5^ec^ and pCj1–HLA-DP5^ec^, were biotinylated with the EZ-Link Maleimide-PEG_11_-Biotin reagent (Thermo Fisher Scientific), according to the manufacturer’s instructions, and then immobilized in a flow cell containing a streptavidin sensor chip (Series S Sensor Chip SA; GE Healthcare), leading to ~1000 resonance units (RU). The SPR measurements were performed on a Biacore T200 (GE Healthcare) instrument by the injection of a series of analytes, TCR(SU3–3)^ec^ diluted with HBS-P buffer (GE Healthcare) to various concentrations (0.3125–10 μM), for 120 s at a flow rate of 10 μl min^−1^ at 20°C, using HBS-P buffer. The final response curves were obtained by subtracting the curve obtained in the absence of the analyte. The equilibrium dissociation constant (*K*_D_) values of NF-pCj1–HLA-DP5^ec^·TCR(SU3–3)^ec^ and pCj1–HLA-DP5^ec^·TCR(SU3–3)^ec^ were calculated with the BIAevaluation software (GE Healthcare).

## Results

### Conserved NF residues of allergenic peptides

**Fig. 1.**
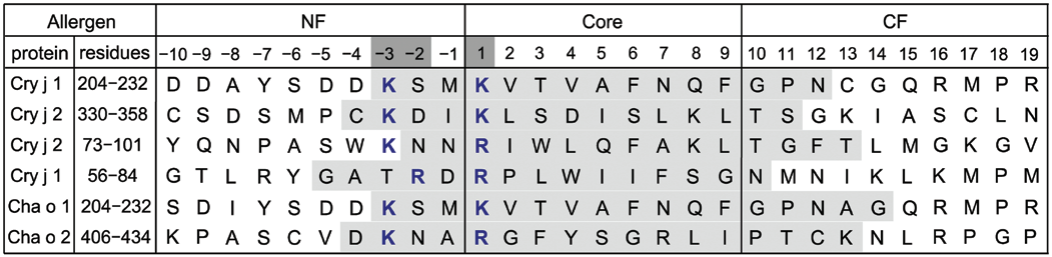
Amino acid sequences of pollen allergenic peptides that bind to HLA-DP5. The amino acid sequences at the peptide positions from P–10 to P19, the NF region (P–4 ~ P–1), the core (P1–P9), and the CF region (P10–P19), of the Cry j 1/2 (4, 5) and Cha o 1/2 peptides that bind to HLA-DP5 (27, 28) were aligned according to the nine-residue core sequence in the pCj1–HLA-DP5 structure (13). The ranges of these 29 residues in the parent proteins are also indicated. Dark gray boxes: conserved peptide positions. Gray boxes: regions corresponding to the isolated HLA-DP5-binding peptides derived from Cry j 1/2 and Cha o 1/2.

Four peptides derived from the major allergens Cry j 1 (4) (residues 61–75 and 211–225) and Cry j 2 (5) (residues 81–95 and 336–350) from *C. japonica* reportedly bind to HLA-DP5 (12, 13). As shown in Fig. 1, the sequences of these HLA-DP5-binding peptides can be aligned well, with respect to the nine core residues (214-KVTVAFNQF-222) of the Cry j 1 epitope. The nine-residue peptide consisting of these core residues (pCj1) was actually bound within the PBG in our previous crystal structure of the pCj1-bound HLA-DP5 molecule (13). For *Chamaecyparis obtusa* (Cha o, Japanese cypress) pollens, the sequences of the two major HLA-DP5-binding peptides (Fig. 1), from the allergens Cha o 1 (27) (residues 211–227) and 2 (28) (residues 412–428), could also be aligned [K. Kino *et al.*, Japan Patent Kokai 2008-195718]. In the NF region of these antigenic peptides, except for Cry j 1 56–84, positions −2 and −3 are occupied by Ser/Asp/Asn and Lys, respectively (Fig. 1). It is remarkable that Lys is conserved at position −3. As for position −2, Ser, Asp, and Asn are classified in common as small hydrophilic amino acid residues, and thus homologous to each other. Therefore, the chemical properties are well conserved at positions −2 and −3 of most of these HLA-DP5-binding peptides related to pollinosis. In this context, in the structures reported so far, the interactions of the NF residues with HLA-II/MHC-II have been observed for the Lys side-chain and/or the main-chain CO and/or NH group(s) at positions −1 and/or −2 in the NF region (15, 16). Therefore, a different type of side-chain-specific (or sequence-specific) interaction(s) of the NF residues was expected for Cry j 1 and HLA-DP5, which prompted us to examine the contributions of the conserved NF residues of Cry j 1 in the HLA-DP5 complex with a longer Cry j 1 peptide. In contrast, no such sequence conservation was observed for the C-terminal flanking (CF) regions of these allergenic peptides.

### Binding of the WT and the S(P–2)E/K(P–3)E mutant of NF-pCj1 to HLA-DP5

**Fig. 2.**
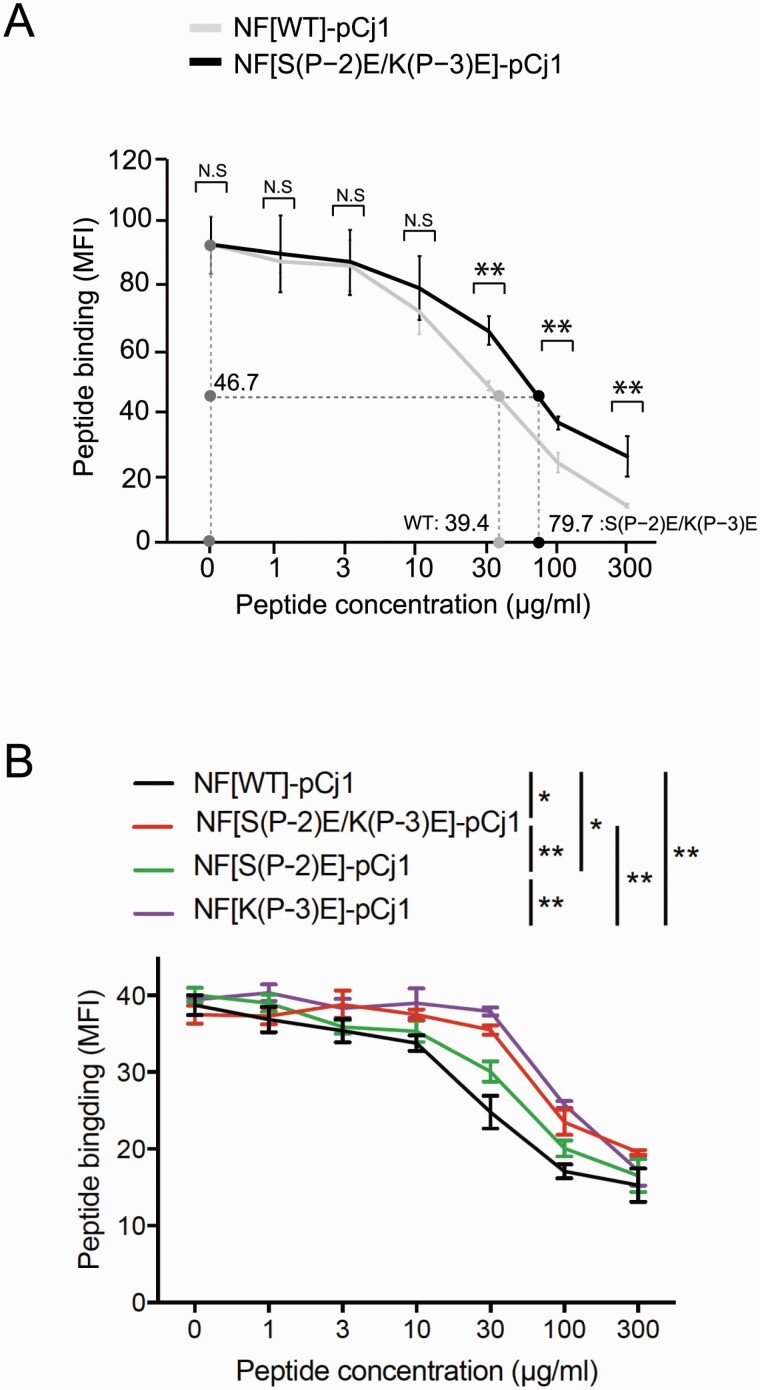
Competition assays. (A) Specific binding of the biotin-conjugated HA1 peptide (ENERTLDFHDSNVKN, 10 μg ml^−1^) to HLA-DP5-expressing HEK293 cells (mean fluorescence intensity, MFI) was assessed by an incubation for 24 h in competition with the wild-type (WT) (gray) and various concentrations of the S(P–2)E/K(P–3)E mutant (black) of C-terminally-flanked NF-pCj1 (0–300 μg ml^−1^), and staining with allophycocyanin-conjugated streptavidin. The half maximal **i**nhibitory concentration (IC_50_) values of the two NF-pCj1 peptides are indicated, and show that the affinity for HLA-DP5 of the WT NF-pCj1 peptide is 2-fold higher than that of the mutant peptide. (B) Competition assays were performed with single mutants S(P–2)E and K(P–3)E as well as the double mutant S(P–2)E/K(P–3)E. Statistical significance: ^*^P < 0.05, ^**^P < 0.01.

We analyzed the binding of Cry j 1 peptides with the WT and double mutant S(P–2)E/K(P–3)E of NF to the full-length HLA-DP5 expressed in HEK293 cells, by a competitive binding assay with the hemagglutinin (HA) peptide (Fig. 2A). As the fluorescent labeling experiments, described below, were performed with NF-pCj1 followed by the three-residue peptide spacer (GPN) and ethylene glycol (EG)-EG-TAMRA, we here used a C-terminally-flanked version of NF-pCj1 (16-residue) and its S(P–2)E/K(P–3)E mutant as the competing peptides, for comparison between the two types of experiments. The WT NF-pCj1 competed with the HA1 peptide well (Fig. 2A). In contrast, the S(P–2)E/K(P–3)E mutant of NF-pCj1 competed less efficiently with the HA1 peptide. Thus, the affinity of NF-pCj1 for HLA-DP5 was about 2-fold higher than that of the NF double mutant. Therefore, the NF residues Ser(P–2) and Lys(P–3) detectably increased the affinity of the peptide for HLA-DP5. We additionally performed HLA-DP5 binding analyses also with single mutants of NF-pCj1, indicating that the NF[K(P–3)E] mutation decreased the binding affinity of pCj1 for HLA-DP5 more than the NF[S(P–2)E] mutation (Fig. 2B).

### Promoted display of NF-pCj1 on APCs by HLA-DP5

**Fig. 3.**
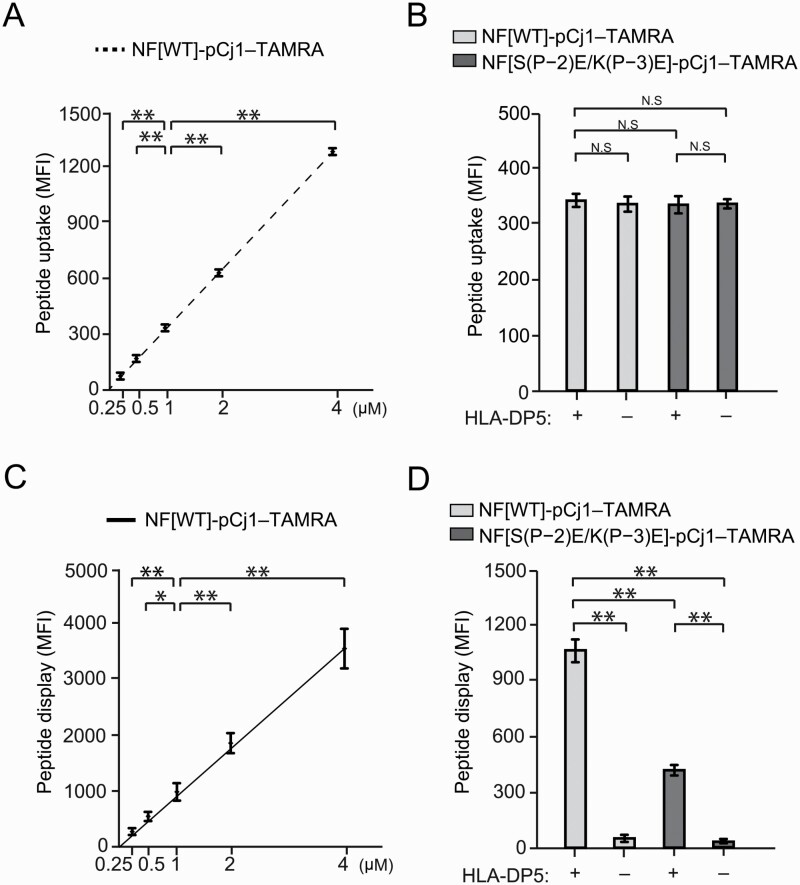
Display of NF-pCj1 on the APC surface. (A and B) Uptake of fluorescent NF-pCj1 into mDC1 cells. (A) and (B) Analyses for MFI values of TAMRA, corresponding to Supplementary Fig. 1A and B, respectively. The MFI values of TAMRA were corrected by calculating the difference between the MFI value in the presence of the TAMRA-conjugated NF-pCj1 and that in its absence. Statistical significance: ^*^P < 0.05, ^**^P < 0.01. (C and D) Fluorescent NF-pCj1 displayed on the surface of mDC1s. (C) Determination of the MFI values of Alexa 647, corresponding to Supplementary Fig. 1C. (D) Analysis of the TAMRA-labeled NF-pCj1 antigen peptide displayed on mDC1 cells expressing HLA-DP5. The MFI values of Alexa647, corresponding to Supplementary Fig. 1D, conjugated to an anti-TAMRA monoclonal antibody for the combinations of NF-pCj1 and HLA-DP5 are shown. The peptides (1 μM) used are the wild-type and the S(P–2)E/K(P−3)E mutant of NF-pCj1. Statistical significance: ^*^P < 0.05, ^**^P < 0.01.

The WT HLA-DP5, encoded by the DPA1*02:02 and DPB1*05:01 genes, was expressed in the mouse antigen-presenting dendritic cell line 1 (mDC1) cells. Subcloned mDC1 cells that express a certain uniform level of the WT HLA-DP5 (Supplementary Fig. 1A) were used for the cell-surface display of peptides through cellular uptake and HLA-DP5-loading. The uptake of the TAMRA-labeled NF-pCj1 peptide (NF-pCj1–TAMRA or NF-pCj1-CF-EG_2_-TAMRA; the CF-EG_2_ region serves as the spacer between the NF-pCj1 and TAMRA) was proportional to its concentration in the medium (Fig. 3A and Supplementary Fig. 1B). In contrast, the uptake of NF-pCj1–TAMRA was not affected by the NF mutation S(P–2)E/K(P–3)E of the peptide (Fig. 3B and Supplementary Fig. 1C). The amounts of surface-displayed NF-pCj1–TAMRA per cell were also monitored with an Alexa 647-labeled anti-TAMRA antibody, and were nearly proportional to the peptide concentration in the range of 0.25–4 μM in the medium (Fig. 3C and D, and Supplementary Fig. 1D and E). Accordingly, under these conditions the amount of the displayed NF-pCj1 is below the level needed to saturate the HLA-DP5 molecules expressed on the surface of the mDC1 cells, and is presumably far smaller than those of the peptides derived from self-proteins. Therefore, even in the presence of an excess of self-peptides, NF-pCj1 can probably be loaded onto the HLA-DP5 molecules in competition with them. The NF mutations S(P–2)E/K(P–3)E of the peptide halved the amounts of the cell-surface displayed peptide, at the same peptide dose/concentration and surface expression level of HLA-DP5 (Fig. 3D and Supplementary Fig. 1E).

### Establishment of NF-pCj1-specific and HLA-DP5-restricted T-cell clones

**Fig. 4.**
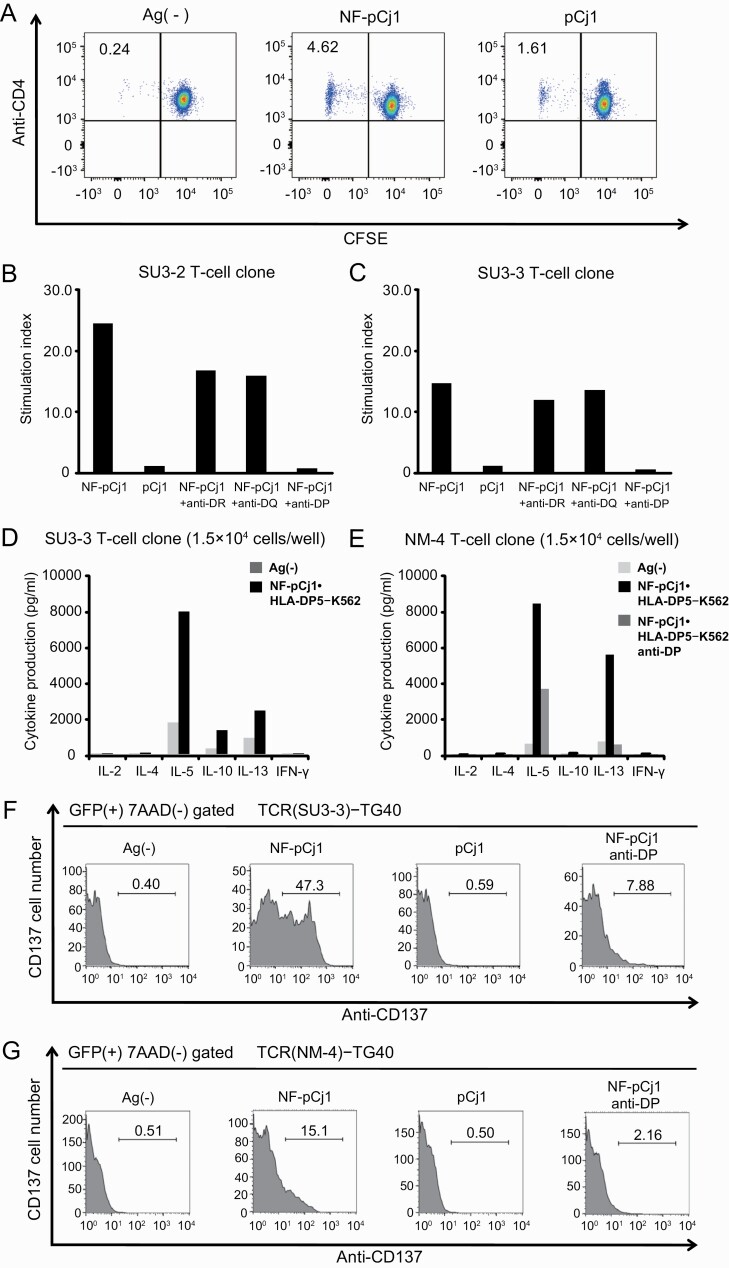
Establishment of T-cell clones. (A) Flow cytometric analysis for the detection of the antigen-specific CD4^+^ T-cell proliferation (7-AAD^−^ CD4^+^ CFSE^dim^), in cells obtained from a representative CP patient who is HLA-DP5-positive. (B and C) The antigen specificities of T-cell clones (SU3–2 and SU3–3) were tested using the ^3^H-thymidine incorporation assay. CD4^+^ T-cell clones were stimulated with NF-pCj1 or pCj1 in the presence of allogenic PBMCs, derived from an HLA-DP5-positive healthy donor. HLA-restriction of T-cell clones was determined by addingvarious monoclonal antibodies (mAbs): anti-HLA-DR mAb (L243, BioLegend), anti-HLA-DQ mAb (SPV-L3, Abcam) or anti-HLA-DP mAb (B7/21, Abcam), at a final concentration of 10 μg ml^−1^. Stimulation index (SI) = counts per minute (cpm) with antigen/cpm without antigen. (D and E) Profiles of cytokines produced by CD4^+^ T-cell clones (SU3–3 and NM-4). CD4^+^ T-cell clones (1.5 × 10^4^ cells per well) were cultured with peptide-pulsed HLA-DP5-K562 cells (1.0 × 10^5^ cells per well) as APCs in 96-well plates. After 24 h, culture supernatants were collected and cytokine levels were measured using the Bio-Plex system (Bio-Rad). (F and G) GFP-transfected TCR(SU3–3)-TG40 cells and TCR(NM-4)-TG40 cells (1.0 × 10^5^ cells per well) were cultured with 10 μg ml^−1^ of NF-pCj1 or pCj1 for 24 h, in the presence of HLA-DP5-positive PBMCs (4 × 10^5^ cells per well). The T-cell surface activation marker CD137 of TCR(SU3–3)-TG40 and TCR(NM-4)-TG40 (GFP^+^ 7AAD^−^ gated) was analyzed by flow cytometry. Data are representative of three independent experiments with similar results.

First, we obtained PBMCs from HLA-DP5^+^ CP patients (SU and NM). From these samples, we isolated many CD4^+^ T cells proliferating in the presence of NF-pCj1 (Fig. 4A center) and established HLA-DP5-restricted T-cell clones (SU3–2, SU3–3, NM-4, and NM-7). Even though the CD4^+^ T cells showed some proliferation in the presence of pCj1 (Fig. 4A right), it was impossible to maintain the cell growth required to establish HLA-DP5-restricted CD4^+^ T-cell clones reactive to pCj1. These T-cell clones proliferated in response to NF-pCj1, as measured by ^3^H-thymidine incorporation, and their responses were specifically blocked by anti-HLA-DP, but not anti-HLA-DR or anti-HLA-DQ mAbs (Fig. 4B and C). Upon stimulation with NF-pCj1 in the presence of HLA-DP5-transfected K562 cells, the SU3–2, SU3–3, NM-4, and NM-7 T-cell clones produced IL-5, IL-10, and IL-13, indicating that these clones have the characteristics of Th2 cells (Fig. 4D and E). We cloned the TCRα and TCRβ genes from these four T-cell clones (Table 1) to establish TCRαβ-expressing mouse T cells, by using TG40, which is a cell-surface TCR^–^, intracytoplasmic CD3^+^, mutant of the 21.2.2 mouse T cell line (29) [TCR(SU3–2)-TG40, TCR(SU3–3)-TG40, TCR(NM-4)-TG40, and TCR(NM-7)-TG40]. These TCR-transfected TG40 cells responded to NF-pCj1, but not to pCj1, as measured by the CD137^+^ cell number in the presence of the HLA-DP5-expressing K562 cells (Fig. 4F and G).

**Table 1.** The variable gene usage of the NF-pCj1–DP5 molecule-reactive TCRs

Cell ID	V alpha	J alpha	AA JUNCTION	V beta	J beta	D beta	AA JUNCTION
(A)							
SU3–2	TRAV8-3*01	TRAJ9*01	CAVGDTGGFKTIF	TRBV7-9*03	TRBJ2-2*01	TRBD2*02	CASSFPGGNTGELFF
SU3–3	TRAV8-3*01	TRAJ34*01	CAVAPTYNTDKLIF	TRBV7-9*03	TRBJ2-2*01	TRBD2*01	CASSSKGGAGELFF
(B)							
NM-4	TRAV16*01	TRAJ48*01	CALRSNFGNEKLTF	TRBV5-1*01	TRBJ2-2*01	TRBD2*02	CASSLVVAGVYTGELFF
NM-7	TRAV12-3*01	TRAJ27*01	CGANAGKSTF	TRBV30*01	TRBJ2-7*01	TRBD2*02	CAWSVMVWGSEGEQYF

### T-cell response promoted by the NF-pCj1 presented on APCs

**Fig. 5.**
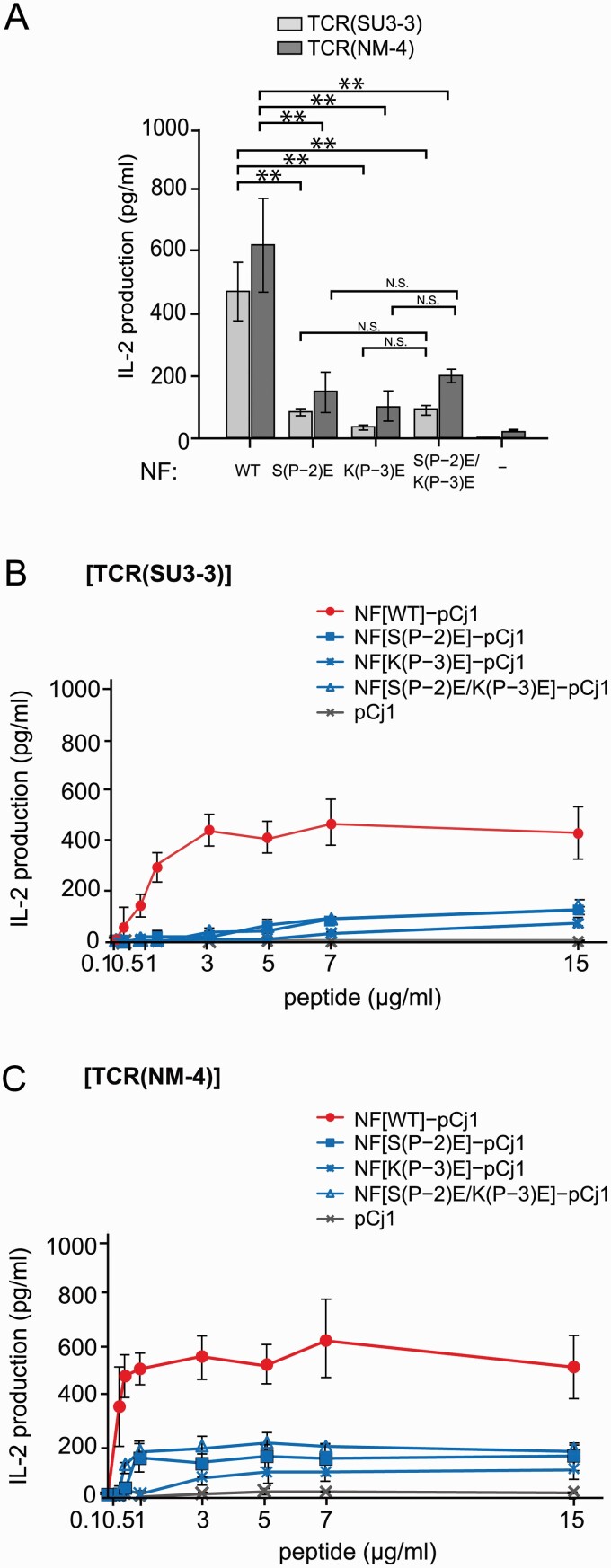
T-cell response to NF-pCj1 presented by mDC1s. (A) Effects of pCj1, NF-pCj1 (7 µg ml^−1^) and its amino acid substitutions on IL-2 production by the TCR-TG40(SU3–3, NM-4) cells cocultured with the HLA-DP5-mDC1s. The antigen peptides were NF[WT]-pCj1, NF[S(P–2)E]-pCj1, NF[K(P–3)E]-pCj1, NF[S(P–2)E/K(P–3)E]-pCj1, and pCj1 (labeled with WT, S(P–2)E, K(P–3)E, S(P–2)E/K(P–3)E, and –, respectively). Statistical significance: ^*^P < 0.05, ^**^P < 0.01. (B and C) Effects of the presence or absence of the NF residue and its amino acid substitutions on the activation of TCR-TG40 cells by antigen-pulsed HLA-DP5-expressing mDC1s. The antigen peptides were NF[WT]-pCj1, NF[S(P–2)E]-pCj1, NF[K(P–3)E]-pCj1, NF[S(P–2)E/K(P–3)E]-pCj1, and pCj1. There are significant differences in the IL-2 production between NF[WT]-pCj1 and the others at antigen doses > 3 µg ml^−1^ (*P* < 0.05).

We then utilized the mDC1 cells stably expressing a defined amount of the WT HLA-DP5, which were used for the cell surface display of peptides described above, to activate the TCRαβ-expressing mouse TG40 cells, TCR(SU3–3)-TG40 and TCR(NM-4)-TG40, with NF-pCj1, as monitored by IL-2 production. In the presence of the HLA-DP5-expressing mDC1s, the TCR(SU3–3)-TG40 and TCR(NM-4)-TG40 cells produced IL-2 in response to NF-pCj1, but not pCj1 (Fig. 5A). Similar positive effects of NF-pCj1 on T-cell activation were observed with a wide range of peptide concentrations (0.5–15 µg ml^−1^) (Fig. 5B and C). The activation of the TCR(SU3–3)-TG40 and TCR(NM-4)-TG40 cells, as monitored by IL-2 production, was dramatically reduced by the NF mutations S(P–2)E, K(P–3)E, and S(P–2)E/K(P–3)E of NF-pCj1 (Fig. 5A–C). Thus, we have clearly demonstrated the remarkable effects of the NF residue mutations on the T-cell activation, using the antigen-presenting mDC1s.

### The intrinsic affinity of NF-pCj1–HLA-DP5^ec^ for the TCR(SU3–3)^ec^

**Fig. 6.**
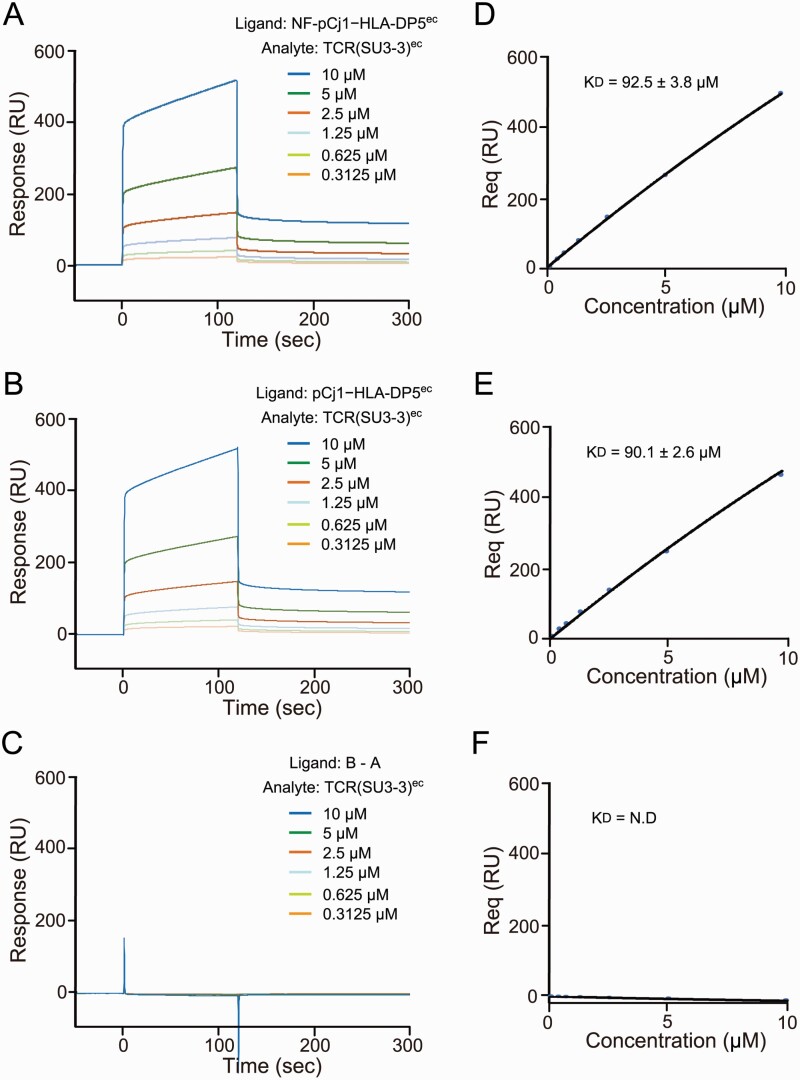
SPR analysis in the presence or absence of NF for TCR^ec^. (A and B) Sensorgrams obtained by the injection of a series of concentrations of TCR(SU3–3)^ec^ over the immobilized NF-pCj1–HLA-DP5^ec^ (A) and pCj1–HLA-DP5^ec^ (B), by a surface plasmon resonance analysis. The dotted bar on the sensorgram indicates the region from which the equilibrium binding responses are derived. RU: resonance unit. (C) Sensorgrams obtained by the subtraction of the RU values of (B) from those of (A). (D–F) Plots of the equilibrium binding responses (Req) against the concentrations of TCR(SU3–3)^ec^ for the sensorgrams in (A) to (C), respectively. The line represents a nonlinear fit of the 1:1 binding model. Data are shown as mean ± standard deviation from three independent experiments.

In several cases, the NF residues of pMHC reportedly interact directly with the TCR CDRs. Therefore, we measured the intrinsic affinities of the peptide-fused forms of the extracellular regions of HLA-DP5, NF-pCj1–HLA-DP5^ec^ and pCj1–HLA-DP5^ec^, for the TCR(SU3–3) extracellular region by SPR. The intrinsic TCR affinity of HLA-DP5 was unaffected by the presence or absence of the NF region (Fig. 6).

## Discussion

In the present study, we found that Ser and Lys at positions −2 and −3, in the NF region of the HLA-DP5-binding peptide (NF-pCj1) from the major allergen Cry j 1 involved in Japanese CP, are well conserved in those identified for other pollinosis allergens. A BLAST search showed that the KSMKVTVAFNQF sequence (the NF sequence KSM followed by the pCj1 core sequence KVTVAFNQF) is highly conserved among the pectate lyase family of proteins widely in gymnosperms, including *Cupressaceae*. The conservation of the NF sequence KSM is probably due to the tertiary folding of the proteins: the KSM sequence forms one turn of 3_10_ helix next to the β strand formed by the KVTVAFNQF sequence in the characteristic β helix tertiary structure (Supplementary Fig. 2, 35). Furthermore, gymnosperms have anemophilous flowers, and many of them are known to cause pollinosis. Therefore, we here discuss the KSMKVTVAFNQF sequence with respect to pollinosis allergen peptides from these plants (rather than HLA-DP5-binding peptides in general).

We determined that the NF residues Ser(P–2) and Lys(P–3) are important for the activation of the NF-pCj1-specific and HLA-DP5-restricted CD4^+^ T-cell clones (SU3–2, SU3–3, NM-4, and NM-7) and the TCRαβ-expressing mouse TG40 cells, TCR(SU3–3)-TG40 and TCR(NM-4)-TG40. The intrinsic TCR affinity of HLA-DP5 was unaffected by the presence or absence of the NF region, whereas the two NF residues increased, by about 2-fold, the affinity of NF-pCj1 for HLA-DP5 and the amount of the peptide displayed on the surface of APCs. These new mechanisms by which the NF region Ser(P–2) and Lys(P–3) contributes to the T-cell activation are presumably among the multiple factors facilitating the creation of immunodominance.

We quantitatively analyzed the effects of the S(P–2)E/K(P–3)E mutations of NF-pCj1 on the antigen peptide presentation by HLA-DP5 and the subsequent T-cell activation. First, the S(P–2)E/K(P–3)E mutations of NF-pCj1 decreased its affinity for HLA-DP5 by about 2-fold, as determined by a new competitive binding assay with the HA1 peptide as the reference. Lys(P–3) appears to contribute to the binding affinity for HLA-DP5 more than Ser(P–2). Second, the S(P–2)E/K(P–3)E mutations of fluorescently labeled NF-pCj1 decreased its amount displayed on the surface of the mDC1s by about 2-fold, at the same levels of peptide uptake into the cell and expression of HLA-DP5 on the cell surface. Correspondingly, the S(P–2)E/K(P–3)E mutation of NF-pCj1 impaired its ability to activate TG40 cells expressing the TCRs cloned from the pollinosis patients, to extents similar to those of the affinity for HLA-DP5 and the cell-surface display of the peptide. Consequently, the NF residues Ser(P–2) and Lys(P–3) are important for the peptide presentation and the T-cell activation.

The PFRs reportedly contribute to the high efficiency of T-cell activation by enhancing the abundance and lifespan of the pMHC on an APC (30). The two NF residues, Ser(P–2) and Lys(P–3), of the antigenic peptide from Cry j 1 (residues 204–232) are conserved as Ser/Asp/Asn and Lys, respectively, in those from the other pollinosis allergens Cry j 2 (73–101/330–358), Cha o 1 (204–232), and Cha o 2 (406–434), except for Cry j 1 (73–101) (Fig. 1). Ser, Asp, and Asn are small hydrophilic amino acid residues that may form hydrogen bonds (hydrogen bond acceptance in common), while Lys residues may form hydrogen bonding and/or electrostatic interactions. These conserved features of the NFR sequence restrict the variety of the peptides that bind to HLA-DP5 for T-cell activation, by 3/20 × 1/20 = 3/400 in theory. A few peptides derived from an antigenic protein can predominantly provoke the T-cell response, in a phenomenon called immunodominance (31). Therefore, the sequence-specific effects of the NFR residues on the T-cell activation may contribute to the strong antigenicity of these HLA-DP5-binding peptides, while the immunodominance of epitopes depends on multiple factors (32). Furthermore, the exceptional Arg(P–2) and Thr(P–3) of Cry j 1 (73–101) (Fig. 1) might functionally substitute for Lys(P–3) and Ser/Asp/Asn(P–2), respectively, based on the assumption that the changes from Lys to Arg and from Ser/Asp/Asn to Thr may shift the positions of the positively-charged and hydrogen-bonding side chains and thereby compensate for the reversed order of the two amino acid residues.

The action mechanisms of the NFR residues Ser(P–2) and Lys(P–3) in T-cell activation are different from those previously reported for the NFR and CFR residues by structural studies (15, 16, 19, 32, 33). First, the crystal structure of murine MHC-II I-A^k^ in complex with a naturally processed peptide from hen egg lysozyme (HEL residues 50–62) revealed that the main-chain C=O groups of Thr(P–1) and Ser(P–2) hydrogen bond with the side-chain Nε2 atom of His81β and the main-chain N–H group of Arg53α, respectively, of I-A^k^ (15). A hydrogen bond between the main-chain C=O of Lys(P–1) and the side-chain Nε2 atom of His81β was also observed in the crystal structure of murine MHC-II I-A^g7^ in complex with the HEL I-A^g7^ epitope (11–25). These interactions involve the main-chain C=O groups, but not the side-chain groups, with MHC-II molecules, indicating the lack of sequence specificity. Furthermore, the main-chain C=O group at position P–1 is alongside the N-terminal residue at position P1 of the core epitope peptide, and is therefore close to the MHC-II surface. This previously identified feature may draw a distinction from the more distal positioning of Lys(P–3) in the NF-pCj1 on HLA-DP5.

Hydrogen-bonding interactions between the NF residues and the TCR have also been reported. The side-chain Nζ group of Lys(P–1) of an epitope peptide from influenza hemagglutinin (HA306–318) bound to HLA-DR1 via hydrogen bonds with the Oε1 and two groups of Glu94α in the CDR3α region of the TCR (33). The main-chain N–H and C=O groups of Gly(P–1) in an α-gliadin-derived peptide (DQ8-glia-a1) bound to HLA-DQ8 via hydrogen bonds with the side-chain Oε group and the main-chain N–H group, respectively, of Gly110α in the CDR3α region of the TCR (19). In addition, the side-chain Nη group of Arg reportedly substituted for the CF residue at positions P10 or P11 of an influenza hemagglutinin epitope peptide (HA305–320) bound to HLA-DR1, and can form a hydrogen bonding/electrostatic interaction with Asp28β of the TCR (18). These interactions of the PFRs from the epitope peptide-bound to MHC (pMHC) with the TCR α chain should increase the affinity of the pMHC complex for the TCR. In contrast, in the present study, we found that the two NF residues Ser(P–2) and Lys(P–3) do not contribute to the affinity of NF-pCj1·HLA-DP5 for the TCRs, as analyzed by SPR.

In summary, Ser(P–2) and Lys(P–3) in the NF region of the antigenic peptide contribute to its presentation by HLA-DP5 on the APC surface and the following activation of T cells. These data provide a demonstration of the N-terminal sequence of the peptide flanking region substantially altering the MHC binding affinity, thus contributing to the T-cell activation. A crystallographic study of the HLA-DP5 and NF-pCj1 complex will clarify the structural mechanisms underlying the strong antigenicity of the epitope peptide. On the other hand, a number of publications described the influence of flanking sequences of MHC class II-binding peptides by fine epitope analyses (18, 34). More structure-based discussion of these epitope analyses would be expected to reveal precise mechanisms of TCR activation by pMHC in general, far beyond pollinosis antigens from gymnosperms.

## Supplementary Material

dxad024_suppl_Supplementary_Figure_S1

dxad024_suppl_Supplementary_Figure_S2

dxad024_suppl_Supplementary_Figure_Legends
