# Supplementary figures and images for: Contributions of the N-terminal flanking residues of an antigenic peptide from the Japanese cedar pollen allergen Cry j 1 to the T-cell activation by HLA-DP5
Source: Int Immunol. 2023 Sep 5;35(9):447–58. doi: 10.1093/intimm/dxad024 (PMC10478803; doi:10.1093/intimm/dxad024)

Supplemental figure 1.

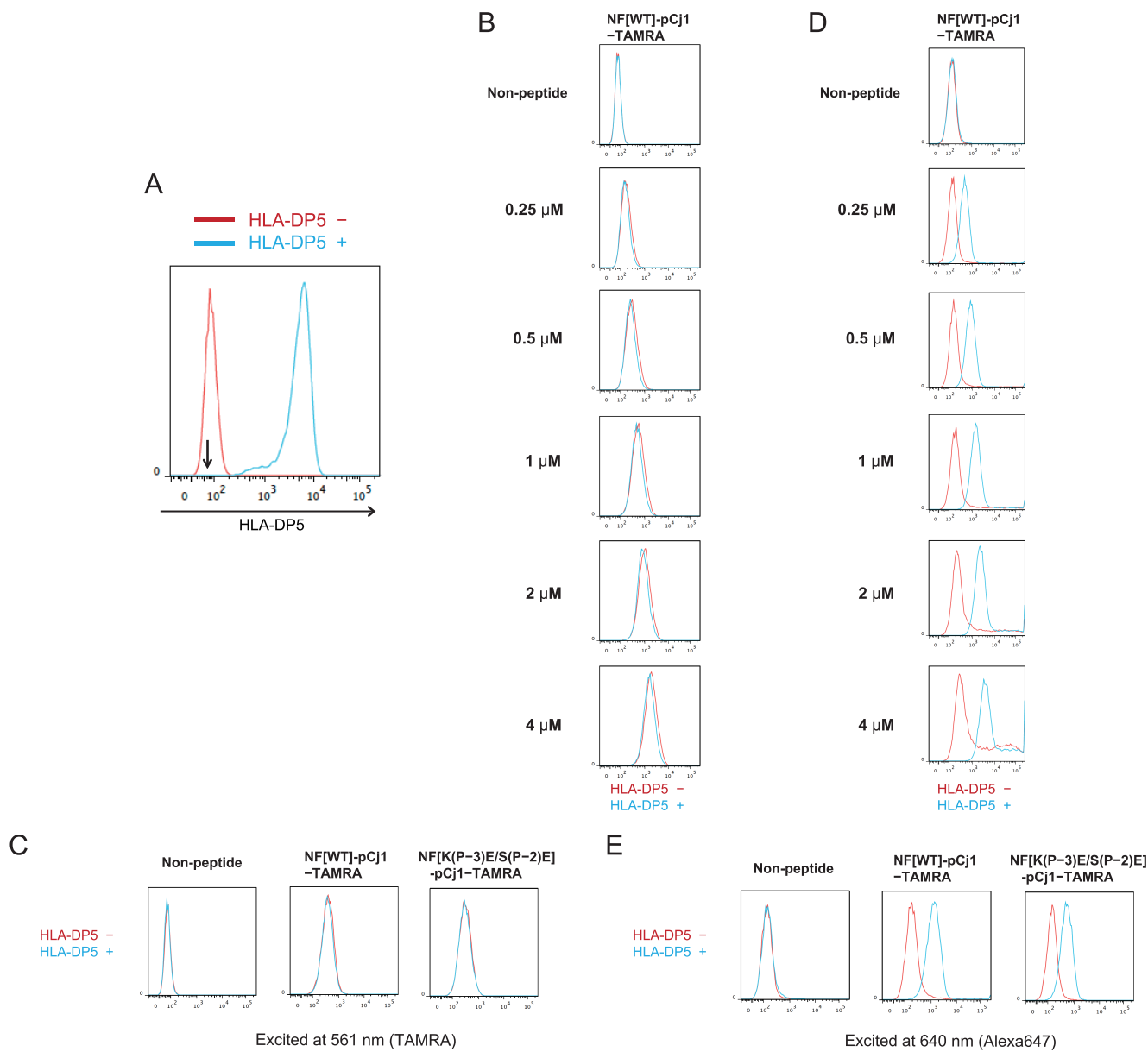

Kusano et al.

Supplement: dxad024_suppl_Supplementary_Figure_S1 [file dxad024_suppl_supplementary_figure_s1.pdf]

## Supplemental figure 2.

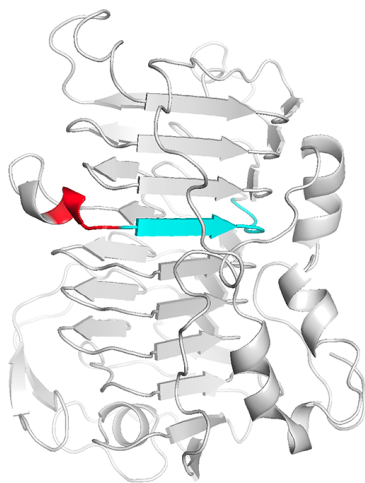

Kusano et al.

Supplement: dxad024_suppl_Supplementary_Figure_S2 [file dxad024_suppl_supplementary_figure_s2.pdf]
