# Supplementary material for: Contributions of the N-terminal flanking residues of an antigenic peptide from the Japanese cedar pollen allergen Cry j 1 to the T-cell activation by HLA-DP5
Source: Int Immunol. 2023 Sep 5;35(9):447–58. doi: 10.1093/intimm/dxad024 (PMC10478803; doi:10.1093/intimm/dxad024)
Supplement: dxad024_suppl_Supplementary_Figure_Legends [file dxad024_suppl_supplementary_figure_legends.docx]

**Supplementary Data**

**Supplementary Figure 1**

Uptake and display of fluorescent NF-pCj1 by mDC1s. (**A**) Expression levels of HLA-DP5 in mDC1s. HLA-DP5α and HLA-DP5β were expressed in mDC1s by transfection. The HLA-DP5 expression on the cell surface was analyzed by flow cytometry. The arrow indicates the peak positions of the control mDC1s not expressing HLA-DP5. Thus, the subcloned transfectant expressed HLA-DP5 at a uniform level on the cell surface. (**B** and **C**) Representative flow cytometry histograms showing the uptake of various concentrations of NF[WT]-pCj1–TAMRA (0.25–4 μM) (**B**), and the uptake of NF[WT]-pCj1–TAMRA or NF[S(P–2)E/K(P–3)E]-pCj1–TAMRA at 1 μM by mDC1s expressing HLA-DP5[WT] (**C**) in the whole cells. In total, 20,000 cells were analyzed by TAMRA excitation at 561 nm for each experiment. The X-axis and Y-axis indicate fluorescence intensity and cell number, respectively. Red and blue lines are parent mDC1s without HLA-DP5 expression and HLA-DP5 stable-cell lines, respectively. All experiments were performed three times with similar results. (**D** and **E**) Representative flow cytometry histograms displaying various concentrations of NF[WT]-pCj1–TAMRA (0.25–4 μM) (**D**), and those of NF[WT]-pCj1–TAMRA or NF[S(P–2)E/K(P–3)E]-pCj1–TAMRA at 1 μM bound to HLA-DP5[WT] (**E**) on the mDC1 surface. In total, 20,000 cells were analyzed by Alexa647 excited at 640 nm for each experiment. The X-axis and Y-axis indicate fluorescence intensity and cell number, respectively. Red and blue lines are parent mDC1s without HLA-DP5 expression and HLA-DP5 stable-cell lines, respectively. All experiments were performed three times with similar results.

**Supplementary Figure 2**

Conformations of the DKSMKVTVAFNQF sequence in the Cry j 1 structure [modeled with AlfaFolds (35) and displayed with PyMOL, https://pymol.org/2/]. The NF sequence DKSM and the pCj1 core sequence KVTVAFNQF are shown in red and cyan, respectively. The pectate lyase catalytic site is located on the back of the β helix.
